# Idiopathic Pulmonary Hemosiderosis: The Great Hemolytic Anemia Mimicker

**DOI:** 10.7759/cureus.40362

**Published:** 2023-06-13

**Authors:** Jaclyn Chan

**Affiliations:** 1 Internal Medicine/Pediatrics, Penn State Health Milton S. Hershey Medical Center, Hershey, USA

**Keywords:** hemolytic anemia, pulmonary hemosiderosis, transfusion dependent anemia, idiopathic pulmonary hemosiderosis, diffuse alveolar hemorrhage, microcytic anemia

## Abstract

Idiopathic pulmonary hemosiderosis (IPH) is a rare, potentially fatal disease characterized by recurrent diffuse alveolar hemorrhage. Presentation varies, and delay in diagnosis and treatment can result in respiratory complications and increased mortality. It is imperative to consider IPH in the differential of a patient presenting with transfusion-dependent microcytic anemia and concomitant pulmonary symptoms.

This case series describes two pediatric patients with persistent severe microcytic anemia despite multiple blood transfusions. Both patients underwent extensive workup for their anemia, and ultimately, their respiratory symptoms led to their diagnosis of IPH. Both were then managed with long-term corticosteroids and had significant clinical improvement.

## Introduction

Idiopathic pulmonary hemosiderosis (IPH) is a rare, potentially life-threatening disease of uncertain etiology characterized by recurrent diffuse alveolar hemorrhage. The hemorrhage causes iron to accumulate in alveolar macrophages, and the accumulation stimulates intracellular ferritin production. The ferritin molecules then store the iron and eventually process the iron into intracellular hemosiderin complexes. This trapped iron is functionally unavailable for hemoglobin synthesis by erythroblasts in the bone marrow, and the depletion of body iron reserves results in an iron deficiency anemia [1]. Patients may have a normal or elevated serum ferritin level despite this iron deficiency secondary to absent bone marrow iron since the measured serum ferritin reflects the trapped hemosiderin iron in the lungs.

IPH can occur at any age, but approximately 80% of cases occur in the pediatric population, and most children present prior to the age of 10 years [2]. The clinical presentation can range from acute onset with hemoptysis and dyspnea to a more subtle, gradual symptomatic anemia. In one retrospective case series of 26 children with IPH, the most common clinical features included cough, breathlessness, fever, hemoptysis, and wheezing, and physical exam findings were most notable for clubbing, hepatomegaly, and splenomegaly [3].

The diagnosis of IPH is challenging as the disease is uncommon, and its presentation can mimic other disorders. Often the hematologic signs and symptoms are more profound than pulmonary symptoms, leading to extensive and unrevealing hematologic evaluation. Following an episode of pulmonary hemorrhage, reticulocytosis may be present, and workup for a suspected hemolytic anemia etiology may be pursued. If hemorrhagic sputum is swallowed, stool may test positive for fecal occult blood, prompting the evaluation of gastrointestinal bleeding. In toddlers, presentation at a time more distant from active hemorrhage can mimic severe iron deficiency anemia with low ferritin, elevated total iron binding capacity, and lack of appropriate reticulocytosis. When prominent pulmonary symptoms are not present, there can be delays in making the diagnosis of IPH. Unfortunately, there is no specific feature pathognomonic to IPH, and diagnosis relies on a high index of suspicion, demonstration of alveolar hemorrhage, and exclusion of other disorders that may cause alveolar hemorrhage.

This article was previously presented as a poster at the Society of Hospital Medicine South Central Pennsylvania Chapter Third Annual Academic Day and Poster Competition (Virtual Conference) on October 17, 2020.

## Case presentation

Case 1

A previously healthy 14-month-old female presented to the emergency department with a two-week history of fever with cough and congestion and a one-week history of emesis. Associated symptoms included weight loss, fatigue, and pallor, and a history significant for excessive milk intake. A complete blood count (CBC) revealed low hemoglobin (Hgb) at 1.7 gm/dL, low hematocrit (Hct) at 7.4%, a low mean corpuscular volume (MCV) of 53.6 femtoliters, and an elevated red cell distribution width (RDW) of 21.4%. The reticulocyte count was elevated at 6.5%, and a chest X-ray was significant for cardiomegaly. The patient was admitted to the pediatric intensive care unit (PICU) for increased work of breathing and tachypnea requiring supplemental oxygen and was transfused with 2 units of packed red blood cells (pRBCs). The patient was suspected to have microcytic anemia secondary to iron deficiency from excessive milk intake and managed as such.

At the follow-up 11 days after hospital discharge, the patient’s Hgb was found to be lower than that at the time of discharge, and her reticulocyte count remained elevated. Over the course of several months she required multiple pRBC transfusions at subsequent follow-up visits with variable responses but her Hgb would always inevitably downtrend, necessitating a cycle of recurrent blood transfusions. This prompted workup for a possible underlying hemolytic process, and labs revealed persistent microcytic anemia, elevated lactate dehydrogenase (LDH), and elevated indirect bilirubin. Serum iron was low-normal and ferritin levels were within normal limits. The patient also had a weakly positive super Coombs test suggestive of a warm hemolytic anemia. A hemolytic anemia of autoimmune etiology was suspected, so the patient was started on a trial of oral corticosteroids.

**Figure 1:**
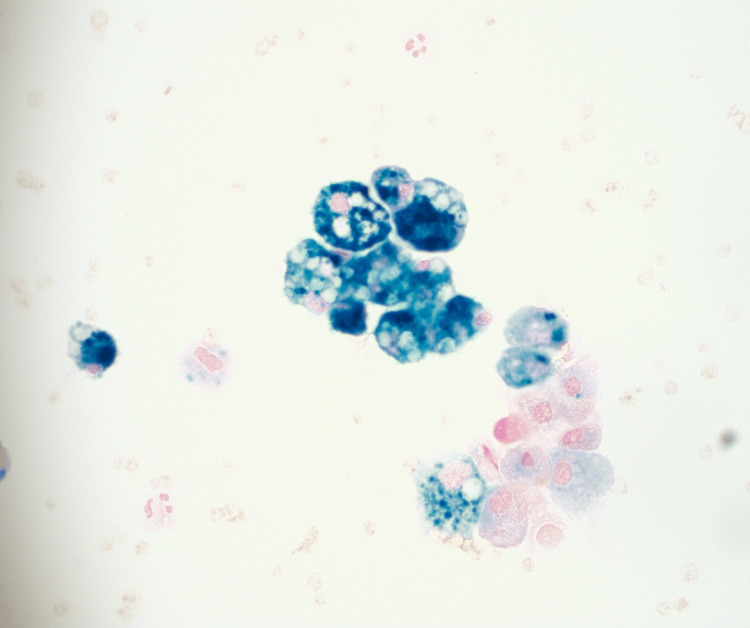
Bronchial washings cytospin stained by Prussian blue (iron stain; magnification 40x). Iron deposition in macrophages is stained dark blue

**Figure 2:**
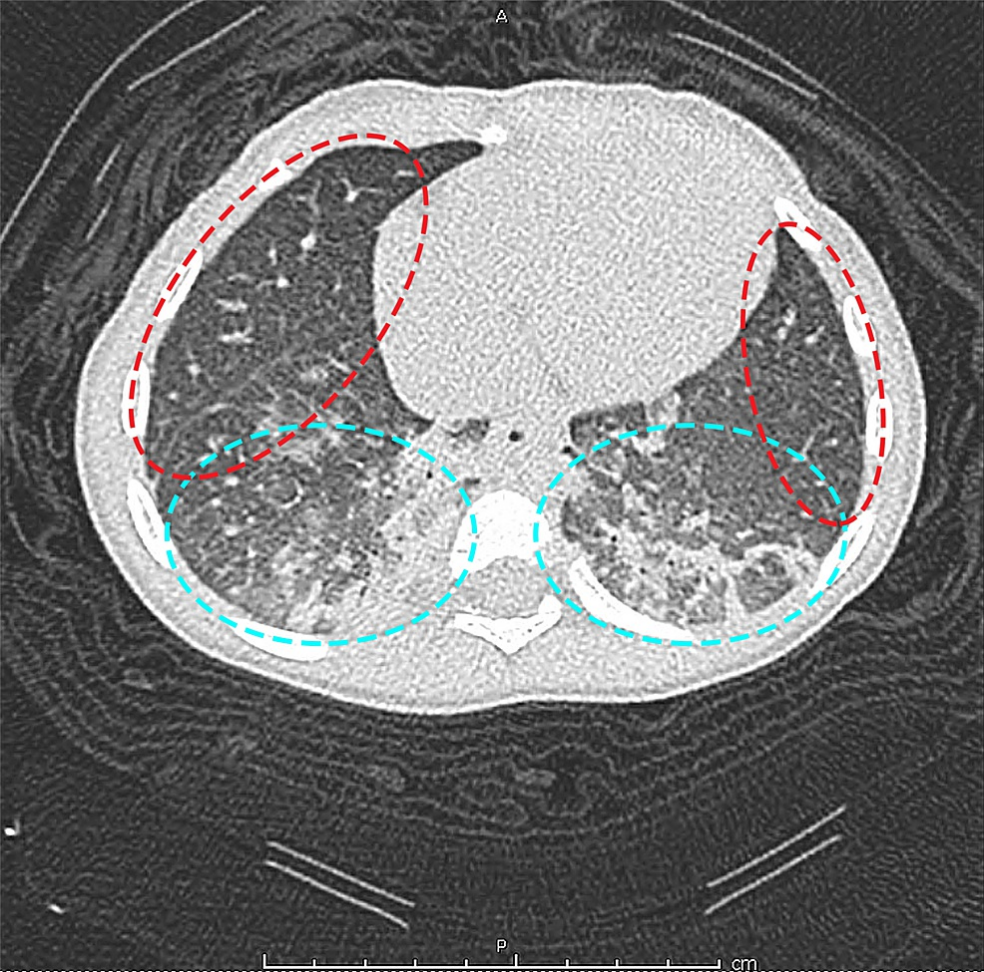
Chest CT without contrast showing bilateral dependent airspace opacities (circled in bright blue) with peripheral groundglass opacity and high-density material (circled in red) consistent with pulmonary hemorrhage

Seven months after the patient’s initial presentation, she was found to have Hgb 4.4 gm/dL during a follow-up visit, prompting a second admission to the hospital for transfusion of pRBCs and initiation of high-dose steroids. At the time of admission, a review of systems was notable for a dry cough and “heavy, noisy breathing”, as described by the patient’s mother. During the admission, the patient had intermittent episodes of tachypnea and increased work of breathing. A chest X-ray showed bilateral hazy opacities throughout both lungs, and a transthoracic echocardiogram showed mild left ventricular enlargement secondary to the anemia but was otherwise unremarkable. The patient underwent further workup with a bone marrow biopsy and aspirate, as well as a bronchoscopy with bronchoalveolar lavage given her respiratory symptoms. The bone marrow report showed marked erythroid hyperplasia, mild dyserythropoiesis, and absent iron stores. Initial gross findings on bronchoscopy were notable for pulmonary hemorrhage, and the bronchoscopy body fluid report revealed numerous hemosiderin-laden macrophages (90% macrophages; see Figure 1). Chest CT without contrast was also consistent with pulmonary hemorrhage (see Figure 2).

A diagnosis of idiopathic pulmonary hemorrhage was made based on bronchoscopy results in conjunction with the patient’s lab findings and clinical presentation, and she was started on oral corticosteroids.

Case 2

A previously healthy 14-month-old female presented to the emergency department with a two-month history of increased sleep, weight loss, a one-week history of nonproductive cough, nasal congestion, and clear rhinorrhea, development of odd eating behaviors including paper, and a three-day history of increased work of breathing and grunting. Her history was also significant for excessive milk intake. In the emergency department, CBC showed low Hgb at 1.4 gm/dL with a low MCV of 62.0 femtoliters, low mean corpuscular hemoglobin (MCH) at 16.0 pg, and an elevated RDW at 25.4%. The reticulocyte count was elevated at 4.5%. The patient was admitted to the PICU and transfused with pRBCs. Her anemia was also suspected to be secondary to iron deficiency and managed as such.

Similar to the patient in Case 1, this patient experienced downtrending Hgb at subsequent follow-up visits requiring recurrent blood transfusions, and she developed elevated indirect bilirubin, inappropriately high reticulocyte count, low haptoglobin, and mild splenomegaly, suggestive of an underlying hemolytic process. Extensive workup for a hemolytic etiology was unrevealing. Six months after initial presentation, the patient developed tachypnea and tachycardia following a blood transfusion that prompted a transthoracic echocardiogram and chest X-ray. The echocardiogram was normal, but the chest X-ray revealed “diffuse, mild to moderate, hazy, increased density of the lungs, which may represent diffuse bilateral pneumonia versus pulmonary hemorrhage versus worsening diffuse interstitial lung disease”. The patient underwent bronchoscopy with bronchoalveolar lavage that revealed hemosiderin-laden macrophages consistent with pulmonary hemorrhage. A bone marrow biopsy and aspirate was also performed at the time and showed erythroid hyperplasia and mild dyserythropoietic changes, with no stainable iron identified.

The patient was diagnosed with idiopathic pulmonary hemosiderosis and started on oral corticosteroids. She was followed up by the Pediatric Pulmonology unit and was eventually weaned and transitioned to inhaled steroids seven months following diagnosis, which she tolerated without recurrence of respiratory symptoms. Following her diagnosis, the patient’s Hgb remained stable and she did not require any further blood transfusions.

## Discussion

In both the cases presented, there was a delay of several months from the time of initial presentation to definitive diagnosis and appropriate treatment, and this challenge was primarily attributed to the fact that the disease was perceived as hemolytic in nature. Additionally, both patients had some degree of iron deficiency attributable to diet and excessive milk intake at the time of presentation, which initially suggested a more common diagnosis such as iron deficiency anemia. They were followed up for their presumed hematologic-related condition, and early in their follow-up, labs were collected to assess hemoglobin trend and patient response to pRBC transfusions. Upon the realization that these patients required multiple blood transfusions for persistently decreasing hemoglobin levels despite adherence to iron supplementation and dietary modifications, the two patients underwent extensive but unrevealing workup for an anemia of hemolytic origin.

Ultimately the patients’ respiratory symptoms prompted workup beyond a hematologic cause, leading to their diagnosis of IPH. Several months had elapsed since initial presentation, and both patients received numerous blood transfusions for symptomatic management that could have very likely been avoided had a definitive diagnosis been made earlier.

The mainstay treatment of IPH focuses on the management of acute alveolar hemorrhage with systemic glucocorticoids, and multiple studies suggest that steroids can prolong survival and improve clinical outcomes in patients with IPH [2,4]. Patients with IPH may also require supportive care such as supplemental oxygen for hypoxemia, as seen in Case 1.

If left untreated, the recurrent alveolar hemorrhage of IPH can lead to various potentially life-threatening complications. Patients may develop hypoxemic respiratory failure requiring ventilatory support. They may also develop fibrotic lung disease as a result of pulmonary hemosiderosis and fibrosis caused by the recurrent alveolar hemorrhage. Thus, it is critical that efforts are made to prevent recurrent pulmonary hemorrhage in patients with IPH.

## Conclusions

Two pediatric cases of IPH were presented to illustrate the difficulty in identifying the diagnosis and the resulting delay in appropriate treatment from the time of initial presentation. In both cases, the patients were initially presumed to have iron deficiency anemia due to excessive milk intake, and when they continued to require multiple blood transfusions despite adequate iron supplementation and diet modifications, workup for a hemolytic etiology was pursued. Consideration of IPH was not included in the differential until both patients displayed significant respiratory symptoms, at which point several months had already elapsed from the time of initial presentation. Fortunately, both patients were promptly started on corticosteroids following their diagnosis of IPH and showed significant clinical improvement and stabilization of their hemoglobin levels.

The goal of this case series is to urge consideration of IPH during the initial workup of patients presenting with persistent severe microcytic anemia suggestive of hemolysis and concurrent pulmonary symptoms in order to minimize the morbidity and mortality associated with complications that arise from the delay in treatment.
